# Comparison of two biosimilarity studies of FKB327 with the adalimumab reference product: randomized phase 1 studies of single-blind, single-dose subcutaneous injection in healthy Japanese male participants

**DOI:** 10.1186/s40360-021-00545-3

**Published:** 2022-01-08

**Authors:** Takuma Yonemura, Rie Yazawa, Miwa Haranaka, Kazuki Kawakami, Masayuki Takanuma, Takumi Kanzo, Dimitris Stefanidis, Yasumasa Arai

**Affiliations:** 1 SOUSEIKAI Sumida Hospital, Tokyo, Japan; 2 SOUSEIKAI Hakata Clinic, Fukuoka, Japan; 3 Fujifilm Kyowa Kirin Biologics Co., Ltd, Tokyo, Japan; 4 Mylan EPD G.K, Tokyo, Japan; 5 Viatris Inc, Bad Homburg vor der Höhe, Germany

**Keywords:** Adalimumab, Bioequivalence, Biosimilar, FKB327, Japanese, Pharmacokinetics

## Abstract

**Background:**

FKB327 has been developed as a biosimilar of the adalimumab reference product (RP). We compared the pharmacokinetics (PK), safety, and immunogenicity of FKB327 with those of the adalimumab RP after a single dose by subcutaneous (SC) injection in Japanese male participants.

**Methods:**

Two randomized, single-blind, single-dose studies were conducted in healthy Japanese male participants to compare PK characteristics between FKB327 and the RP. Study 1 included 130 participants who were randomized in a 1:1 ratio to receive a subcutaneous injection of 40 mg of either FKB327 or the RP into the abdomen. In Study 2, another 130 subjects were randomized in a 1:1 ratio to receive either drug as in Study 1, but the drug administration site was changed to the thigh. The primary PK endpoints of both studies were area under the concentration-time curve from time zero to the last measurable concentration (AUC_0-t_) and maximum serum concentration; area under the concentration-time curve from time zero to 360 h was also evaluated as one of the primary endpoints in Study 1. Biosimilarity in terms of pharmacokinetics was determined if the 90% confidence interval of the mean difference in geometric mean ratio of all primary PK parameters was within the prespecified equivalence criteria (0.80–1.25). Immunogenicity and safety were also evaluated as secondary endpoints.

**Results:**

The serum concentration-time profiles were comparable between the FKB327 and the RP treatment groups in both studies. Primary PK parameters were within the prespecified bioequivalence range in Study 2, although AUC_0-t_ was slightly outside the upper side of the range in Study 1. No differences in safety profile were observed in these studies. The incidence of anti-drug antibodies (ADAs) and impact of ADAs on PK profile were similar among the treatment groups in both studies.

**Conclusion:**

Biosimilarity between FKB327 and the RP after a single 40-mg SC injection was confirmed in healthy Japanese male participants by modifying the study design.

**Trial registration:**

jRCT2071200058 (https://jrct.niph.go.jp/en-latest-detail/jRCT2071200058, https://rctportal.niph.go.jp/en/detail?trial_id=jRCT2071200058) and jRCT2071200057 (https://jrct.niph.go.jp/en-latest-detail/jRCT2071200057, https://rctportal.niph.go.jp/en/detail?trial_id=jRCT2071200057). Retrospectively registered 25/11/2020.

**Supplementary Information:**

The online version contains supplementary material available at 10.1186/s40360-021-00545-3.

## Background

Adalimumab, a recombinant human immunoglobulin G1 monoclonal antibody, binds specifically to human tumor necrosis factor (TNF)-α and neutralizes the biological function of TNF [1–3]. Adalimumab has been approved as a treatment for rheumatoid arthritis, psoriasis, inflammatory bowel disease, and other chronic immune-mediated inflammatory diseases in the United States, the European Union, Japan, and other countries worldwide under the trade name Humira®, hereafter referred to as the reference product (RP) [1–3]. The RP is administered subcutaneously every 2 weeks, with a fixed dosage in adults [2]. However, high pharmacokinetic (PK) variability of the drug has been reported after subcutaneous (SC) injection [3, 4]. Anti-drug antibodies (ADAs) were detected by high-sensitivity ADA assay methods used in recent biosimilar studies [5, 6], although a low ADA-positive ratio was reported in initial studies using a conventional ADA assay method [7]. Given the wider PK variability of the RP, healthy male participants comprising a more homogeneous population without immunosuppressive conditions are considered a more sensitive population in which to assess the PK similarity of its biosimilar product [8–10].

FKB327 is a monoclonal antibody produced in the Chinese hamster ovary cells transfected with complementary DNA encoding the heavy and light chain of the RP and has been developed as a biosimilar of the RP [2]. It has been reported that, along with physicochemical and biological similarities, the PK of FKB327 is similar to that of the US-approved and EU-licensed RP [11, 12]. In addition, FKB327 showed similar efficacy and safety compared to the RP in patients with rheumatoid arthritis [13, 14].

To evaluate the PK similarity of FKB327 compared with the RP in a Japanese population, 2 biosimilarity studies were performed in accordance with Japanese biosimilar guidance, which requires Japanese study participants to be included in either a comparative PK or comparative clinical efficacy study [15]. That is, because the initial FKB327–004 study (Study 1) did not fully confirm the biosimilarity with reference to the general bioequivalence criteria of all primary PK endpoints, the FKB327–006 study (Study 2) was designed to confirm biosimilarity between the study drugs by modifying the study design. Here, we report the outcomes of the 2 biosimilarity studies comparing FKB327 with the RP in healthy Japanese male participants and evaluate the impact of the study design modification on study outcomes.

## Methods

### Subjects

Study 1 (protocol number FKB327–004, jRCT2071200058; 25/11/2020) was conducted at Souseikai Sumida Hospital in Japan from December 2015 to June 2016; Study 2 (protocol number FKB327–006, jRCT2071200057; 25/11/2020), with almost the same study design as Study 1, was conducted at Souseikai Hakata Clinic and Souseikai Sumida Hospital in Japan from July 2017 to November 2017. Both FKB327–004 and − 006 studies were approved by the Hakata Clinic Institutional Review Board, Fukuoka, Japan, under the committee’s reference numbers: 1570BS (18/12/2015) and 1570BS-2 (16/06/2017), respectively, and the studies were conducted in compliance with the International Ethical Guidelines for Biomedical Research Involving Human Subjects, International Conference on Harmonisation Good Clinical Practice (ICH-GCP) guidelines, the Declaration of Helsinki, and local laws. All participants provided written informed consent prior to initiation of the study, in accordance with ICH-GCP.

A total of 130 healthy male participants, aged 20 to 44 years, with a body mass index ≥18.5 kg/m^2^ and < 25.0 kg/m^2^ at screening were enrolled in each study. Participants were excluded from these studies if they had an infection (bacterial, viral, fungal, or parasitic) ≤28 days prior to administration of the study drugs; tested positive for an infectious disease, including hepatitis B surface antigen/antibody, hepatitis C virus antigen/antibody, HIV antigen/antibody, or tuberculosis; had a history of cancer; participated in another clinical study within the past 4 months; or were previously treated with the RP.

### Study design

Studies 1 and 2 were Phase 1 randomized, active-controlled, single-blinded, single-dose, parallel-group studies designed to compare the PK similarity of FKB327 and the RP in a Japanese population. Eligible participants were admitted to the investigational site 1 day before dosing (Day − 1) and underwent predose examinations. The eligible participants were randomized in a 1:1 ratio to either of the treatment groups (65 participants each planned in the FKB327 and RP groups) by weight (< 65 kg and ≥ 65 kg) according to the computer-generated randomization list. Trial site was included as a stratification factor in Study 2 because the study was conducted at 2 sites. Randomisation was stratified with with each cohort using a block size of four. Participants received a 40-mg single dose of either FKB327 or the RP in accordance with the study drug allocation by SC injection in the abdomen (Study 1) or in the thigh (Study 2) in a blinded manner by masking the injection during the studies. After completing specified examinations and assessments on Day 9, participants were discharged if no clinical abnormalities requiring hospitalization for follow-up were observed. Thereafter, the participants revisited the trial site on specified days between Days 16 and 65 for PK and safety assessments.

### Assessments

Blood samples for PK analysis were taken prior to dosing and at 4, 12, 24, 36, and 48 h, and on Days 4, 5, 6, 7, 8, 9, 16, 23, 30, 37, 44, 51, and 65 after dosing. Serum concentrations of FKB327 and the RP were determined using a validated immunoassay on an electrochemiluminescence (ECL) platform using 96-well Meso Scale Discovery (MSD) high-bind plates coated with TNF-α [12]. The lower limit of reliable quantification was 100 ng/mL. ADA activity as an immunogenicity assessment for FKB327 and the RP were analyzed at pre-dose and at Days 1, 16, 30, and 65 after a single dose of the study drug in both studies. A highly sensitive ECL bridging format (MSD) with acid dissociation to increase drug tolerance was used for ADA assessments and ADA titer [13, 16]. ADA titer was defined as low (≤25th percentile), moderate (between the lower and upper percentile), or high (≥75th percentile). The percentage of patients with neutralizing ADAs (NAbs) was determined by sensitive competitive ligand binding [17]. Mean serum drug concentration-time profiles by ADA categorical titers at the last sampling time point were analyzed to evaluate the impact of ADA on the PK of FKB327 and the RP.

The safety of FKB327 compared with the RP was assessed through the reporting of adverse events (AEs), physical examinations, vital sign measurements, electrocardiograms (ECGs), and clinical laboratory safety tests of blood and urine, which were measured at trial sites. Treatment-emergent AEs (TEAEs) were summarized by system organ class and preferred term using the Medical Dictionary for Regulatory Activities in both studies (version 19.0 in Study 1, version 20.1 in Study 2). In Study 1, as local tolerability checks, injection-site reaction and injection-site pain were also evaluated immediately after dosing and at 0.5, 1, 12, and 24 h after SC dosing, using a visual analog scale (VAS) for pain (0 = no pain; 100 = intolerable pain) [18].

### Investigational product

In both studies, participants were randomized 1:1 (each treatment group, *n* = 65) to receive 40 mg of either FKB327 (supplied by Fujifilm Kyowa Kirin Co., Ltd., Tokyo, Japan) or US-approved RP, (citrate-containing [2]) administered via prefilled syringe (both at 40 mg/0.8 mL). In Study 1, participants received a single SC injection of 40 mg of FKB327 or the RP into the abdomen. In Study 2, participants received the same SC dose of FKB327 or the RP, with the injection site changed to the thigh, with the expectation of less PK variability [19]. All participants in both studies received the study drug under single-blinded conditions to evaluate safety without bias.

### Statistical analysis

All participants who received the study drug were included in the safety analysis set. The sample sizes for these studies were estimated based on previous bioavailability or bioequivalence studies with the RP and/or FKB327 [5, 12, 20], in which the coefficient of variation (CV) of maximum concentration (C_max_) and area under the concentration-time curve from time zero to the last measurable concentration (AUC_0-t_) of FKB327 and the RP were reported to be approximately 40%. A total of 130 participants (65 in each treatment group) were enrolled so that the 90% confidence interval (CI) for the mean difference of primary PK values would be within the bioequivalence criteria with a power of 80%. PK parameters were calculated using noncompartmental analyses (WinNonlin, Pharsight; St. Louis, Missouri, USA) for all participants with an evaluable FKB327 or RP serum concentration-time profile, and this population was used for the primary analysis of biosimilarity. If data were missing in the analyses shown in this section, the data were treated as missing without imputation. Concentration below the lower limit of quantification was treated as 0 ng/mL in the PK analyses.

PK similarity was evaluated by comparing the 90% CIs for the geometric mean ratios for the primary PK endpoint of C_max_ and AUC_0-t_ between treatments using bioequivalence criteria of 0.80 to 1.25 in both studies; area under the concentration-time curve from time 0 to 360 h was also evaluated as one of the primary endpoints in Study 1. Area under the concentration-time curve from time 0 to infinity (AUC_0-∞_) and half-life were evaluated as secondary PK parameters.

In Study 1, the primary hypothesis was evaluated using analysis of variance (ANOVA) as a prespecified analysis method without adjustment by protein content of drugs used in the study. In addition, an analysis of covariance (ANCOVA) was used, including ADA categorical titers at the last sampling time point in the model. By contrast, in Study 2, primary PK analyses were performed using a protein content–corrected serum drug concentration (RP/FKB327 = 0.98), and primary PK similarity analysis was performed by an ANCOVA that included trial site, body weight, and age in the model due to the potential impact of one of the primary PK parameters that was slightly outside the bioequivalence criteria in Study 1 [12]. In addition, the ANOVA was performed as post hoc analysis, using measured serum concentration (no correction by protein content of drugs) in Study 2 to compare the PK similarity results from Study 1. The safety of FKB327 compared with that of the RP was evaluated through descriptive summaries of AEs, clinical laboratory tests, physical examinations, vital signs, ECGs, and incidence of ADAs and NAbs. Datasets and outputs were produced using SAS® version 9.1 (Cary, NC, USA) or higher.

## Results

### Participant disposition and demographics

**Fig. 1:**
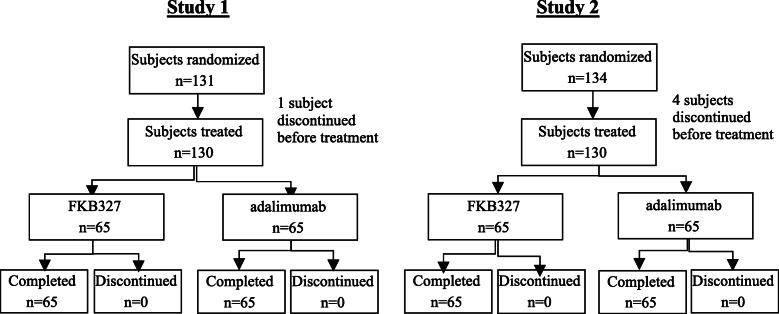
Participant disposition in Study 1 and Study 2

Participant disposition in Study 1 and Study 2 is presented in Fig. 1.

In Study 1, of 131 randomized participants, 1 participant in the FKB327 group withdrew consent;130 participants (65 in each treatment group) received study drugs, with all participants completing the study. In Study 2, of 134 randomized participants, 1 participant each in the FKB327 and RP groups withdrew consent, and 2 participants in the RP group were dropped from the study due to AEs before study drug dosing; 130 participants (65 in each group) received study drugs and all 130 participants completed the study. Thus, all subjects who received study drugs in Study 1 and Study 2 were included in the safety analysis set and PK analysis set. A summary of baseline characteristics in both studies is provided in Table 1. Baseline characteristics were well balanced between treatment groups in both studies.

**Table 1:** Participant demographics and baseline characteristics in Study 1 and Study 2

		Study 1		Study 2	
		FKB327 *N* = 65	RP *N* = 65	FKB327 *N* = 65	RP *N* = 65
Age	Mean (SD)	28.1 (6.96)	28.2 (8.12)	26.5 (7.29)	27.1 (7.56)
BMI	Mean (SD)	21.14 (1.581)	21.15 (1.549)	21.23 (1.905)	21.20 (1.695)
Weight	Mean (SD)	61.92 (6.175)	62.56 (6.412)	61.74 (7.087)	62.09 (6.269)
	< 65 kg (n [%])	45 (69.2)	44 (67.7)	46 (70.8)	44 (67.7)
	≥65 kg (n [%])	20 (30.8)	21 (32.3)	19 (29.2)	21 (32.3)

*BMI* Body mass index; *RP* Reference product; *SD* Standard deviation

### Pharmacokinetics

**Fig. 2:**
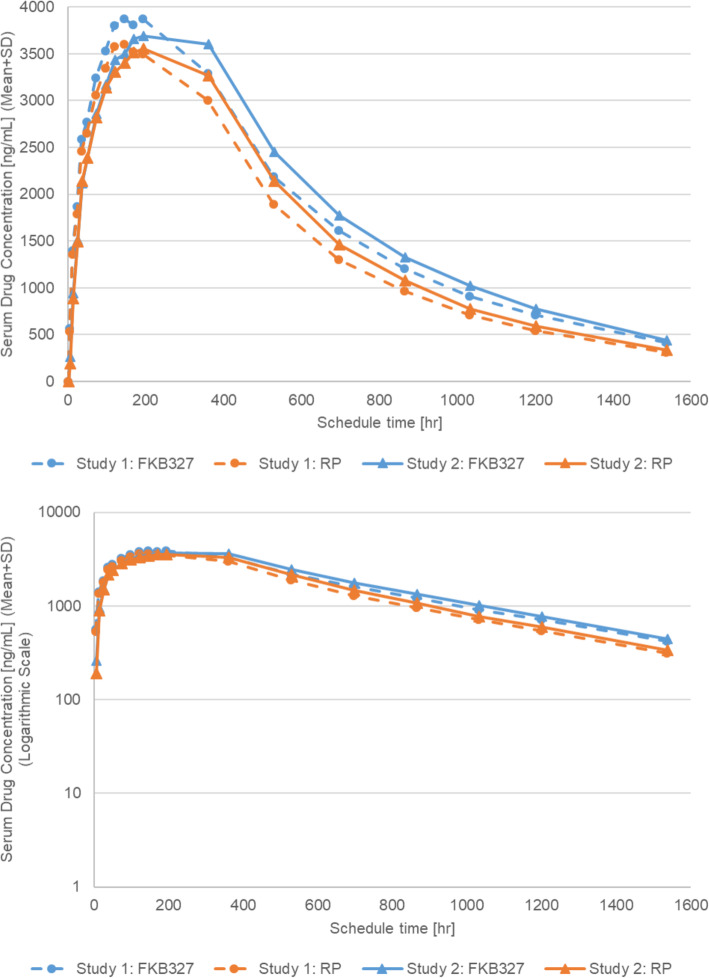
Mean serum concentration-time plots of FKB327 and reference product in participant disposition in Study 1 and Study 2. RP, reference product

The mean serum drug concentration-time profiles of the study drugs following a single SC administration of FKB327 and RP were comparable in Study 1 and Study 2 (Fig. 2).

The mean serum drug concentration was reached as C_max_ slightly earlier in both treatment groups in Study 1 with abdominal administration than in Study 2 with thigh administration. There was no difference in the C_max_ between the FKB327 group and the RP group in both studies; C_max_ values (geometric mean [geometric CV]) in the FKB327 and the RP groups were 3920 (25.2) and 3650 (29.5) ng/mL, respectively, in Study 1, and 3920 (19.1) and 3710 (17.5) ng/mL, respectively, in Study 2, which showed lower PK variability of both drugs in Study 2 (thigh administration) than in Study 1 (abdominal administration) (Table 2).

**Table 2:** Summary of pharmacokinetic parameters in Study 1 and Study 2

	Geometric Mean Parameter Estimate (Geometric CV)^a^							
	Study 1				Study 2			
	FKB327 *N* = 65		RP *N* = 65		FKB327 *N* = 65		RP *N* = 65	
	n	value	n	value	n	value	n	value
C_max_ (ng/mL)	65	3920 (25.2)	65	3650 (29.5)	65	3920 (19.1)	65	3710 (17.5)
AUC_0-t_ (h•ng/mL)	65	2,540,000 (36.7)	65	2,180,000 (41.3)	65	2,620,000 (38.5)	65	2,350,000 (33.7)
AUC_0-∞_ (h•ng/mL)	55	2,770,000 (37.5)	58	2,380,000 (41.9)	56	2,830,000 (40.6)	58	2,550,000 (35.9)
AUC_0–360_ (h•ng/mL)	65	1,170,000 (26.3)	65	1,070,000 (32.2)	65	1,130,000 (21.3)	65	1,080,000 (20.2)
T_1/2_ (h)	55	330.487 (53.7)	58	288.644 (58.2)	56	281.084 (60.8)	58	275.219 (61.8)

^a^Measured values (no correction for protein content of drugs) were used for the analysis in Study 1 and Study 2

*AUC*_*0-∞*_ Area under the concentration-time curve from time zero to infinity; *AUC*_*0–360*_ Area under the concentration-time curve from time zero to 360 h; *AUC*_*0-t*_ Area under the concentration-time curve from time zero to the last measurable concentration; *C*_*max*_ Maximum serum concentration; *CV* Coefficient of variation; *RP* Reference product; *T*_*1/2*_ Half-life

The variability of serum drug concentration in the FKB327 and the RP groups was low at Day 16, with CVs of 19.4 and 23.3%, respectively, in Study 1, and 16.4 and 17.5%, respectively, in Study 2. Thereafter, the CVs gradually increased to a similar degree in both groups until the last blood sampling time of Day 65, when CVs were 89.3 and 105.5%, respectively, in Study 1, and 96.8 and 108.4%, respectively, in Study 2 (Supplemental Table 1).

In the comparison of PK parameters, the C_max_ and AUC_0-t_ of the FKB327 treatment group were slightly higher than those of the RP treatment group in both studies (Table 2). For interindividual variabilities (ie, geometric CV values), the variability of C_max_ in Study 1 was slightly greater in both the FKB327 and the RP treatment groups than in Study 2; however, no trend in variability of any other PK parameters was observed between studies. Although AUC_0-t_ and AUC_0-∞_ exceeded the upper limit of the bioequivalence range by ANOVA in Study 1, the primary similarity analysis of 90% CIs for the geometric least squares means of primary PK parameters (C_max_ and AUC_0-t_) and the other secondary PK parameters by ANCOVA using corrected values were fully contained within the bioequivalence criteria of 0.80 to 1.25 in Study 2. The comparison of primary PK similarity assessment by ANOVA and ANCOVA using measured values (no correction for protein content of drugs) in Study 1 and Study 2 is shown in Table 3.

**Table 3:** Summary of pharmacokinetic similarity analysis in Study 1 and Study 2

	Ratio of geometric LS mean (90% CI)			
	Study 1		Study 2	
	FKB327 / RP		FKB327 / RP	
Parameters	ANOVA	ANCOVA^a^	ANOVA	ANCOVA^a^
C_max_	1.07 (0.99, 1.16)	1.06 (0.99, 1.13)	1.05 (1.00, 1.11)	1.05 (1.01, 1.10)
AUC_0-t_	1.17 (1.05, 1.30)^b^	1.15 (1.04, 1.28) ^b^	1.11 (1.01, 1.23)	1.12 (1.01, 1.23)
AUC_0-∞_	1.16 (1.03, 1.31) ^b^	1.16 (1.04, 1.29) ^b^	1.11 (0.99, 1.24)	1.12 (1.00, 1.25)^b^
AUC_0–360_	1.10 (1.01, 1.19)	1.08 (1.01, 1.16)	1.04 (0.98, 1.10)	1.04 (0.99, 1.10)
T_1/2_	1.14 (0.97, 1.35)^b^	1.14 (0.97, 1.34)^b^	1.02 (0.86, 1.22)	1.02 (0.85, 1.22)

^a^ANCOVA: Body weight and age in both Study 1 and Study 2, and site added in Study 2 as covariates

^b^Data did not meet prespecified equivalence criteria of 0.80 to 1.25

*ANCOVA* Analysis of covariance; *ANOVA* Analysis of variance; *AUC*_*0-∞*_ Area under the concentration-time curve from time zero to infinity; *AUC*_*0–360*_ Area under the concentration-time curve from time zero to 360 h; *AUC*_*0-t*_ Area under the concentration-time curve from time zero to the last measurable concentration; *C*_*max*_ Maximum serum concentration; *RP* Reference product; *T*_*1/2*_ Half-life

In Study 2, the biosimilarity between FKB327 and the RP in healthy Japanese male participants was confirmed, and equivalence for primary PK parameters was maintained following the use of analysis methods with/without covariates and correction for protein content of drugs used in the study.

### Safety

In Study 1, 38.5% of participants in the FKB327 group and 44.6% in the RP treatment group experienced ≥1 TEAE; in Study 2, 30.8 and 47.7% of participants experienced ≥1 TEAE in the FKB327 and RP groups, respectively. In Study 2, 1 participant in the RP treatment group experienced a treatment-emergent serious adverse event (TESAE; frontal bone fracture/skull fracture) 41 days after dosing, was hospitalized for 9 days, and resolved with medication. The TESAE was considered unrelated to the study drug. Neither TEAEs that were related to the study device nor device complaints were reported in studies.

The most frequently reported TEAEs in the FKB327 and the RP groups included injection-site reaction, which was slightly less prevalent in the FKB327 group compared with the RP group (Study 1, 4.6% vs 13.8%; Study 2, 13.8% vs 20.0%), and headache, where the incidence was slightly higher in the FKB327 group than in the RP group (Study 1, 10.8% vs 6.2%; Study 2, 1.5% vs 0%; Table 4).

**Table 4:** Treatment-emergent adverse events reported for ≥3 participants in either treatment group in Study 1 and Study 2

	Study 1		Study 2	
	FKB327 *N* = 65	RP *N* = 65	FKB327 *N* = 65	RP *N* = 65
Participants with ≥1 TEAEs, n (%)	25 (38.5)	29 (44.6)	20 (30.8)	31 (47.7)
Participants with ≥1 TESAEs, n (%)	0	0	0	1 (1.5)
Participants in either group with ≥3TEAEs				
Injection-site reaction, n (%)	3 (4.6)	9 (13.8)	9 (13.8)	13 (20.0)
Nasopharyngitis, n (%)	9 (13.8)	8 (12.3)	3 (4.6)	8 (12.3)
Headache, n (%)	7 (10.8)	4 (6.2)	1 (1.5)	0
Rash, n (%)	1 (1.5)	0	2 (3.1)	3 (4.6)
Diarrhea, n (%)	1 (1.5)	1 (1.5)	2 (3.1)	3 (4.6)
Alanine aminotransferase increased, n (%)	0	0	3 (4.6)	2 (3.1)
Arthralgia, n (%)	0	0	3 (4.6)	1 (1.5)

*RP* Reference product; *TEAE* Treatment-emergent adverse event; *TESAE* Treatment-emergent serious adverse event

No trend in differences was observed for other TEAEs, such as nasopharyngitis, rash, and diarrhea. White blood cell, neutrophil, and monocyte counts decreased between Day 2 and Day 9, and recovered between Day 30 and Day 65 in both treatment groups, and were similar for FKB327 and the RP in both studies. No clinically relevant changes or trends in clinical laboratory tests, ECG, or vital signs were observed during these studies. In Study 1, the median injection-site pain immediately after administration evaluated by 0- to 100-mm VAS scores was lower in the FKB327 group (2.0; range, 0–30) than in the RP group (22.0; range, 0–87), although scores varied widely among participants. VAS pain scores at 0.5 h after dosing were similar between treatment groups, with median scores of 0 (range, 0–13) and 0 (range, 0–33) in the FKB327 and the RP groups, respectively; the median VAS score was 0 in both groups at all other time points from 1 to 24 h after dosing.

### Immunogenicity

At each sampling time point, the prevalence of ADA activity was consistent between the treatment groups. Prior to administration of FKB327 or the RP, 7.7% of participants in the FKB327 group and 4.6% in the RP group showed positive ADA activity in Study 1, and 7.7% of participants showed positive ADA activity in both treatment groups in Study 2. Thereafter, the number of positive cases increased, with 40.0 and 38.5% of participants in the FKB327 and the RP groups, respectively, testing positive for ADA activity at Day 16 postdose in Study 1, and 69.2 and 60.0% of participants in the FKB327 and the RP groups, respectively, testing positive for ADA activity in Study 2. ADA activity at Day 30 or later was observed in most participants in both studies, with 98.5 and 100% of participants in the FKB327 and the RP groups, respectively, testing positive at Day 65 in Study 1 and 100% in both treatment groups testing positive for ADA activity in Study 2. Distribution of ADA titer category at the last sampling time point was similar between the FKB327 and the RP groups in Study 1 and Study 2 (Supplemental Table 2).

The proportion of positive NAbs at the last sampling time point was comparable between the treatments (Study 1, 85.9 and 90.8% in the FKB327 and the RP groups, respectively; Study 2, 92.3 and 87.7%, respectively). NAbs were detected in the majority of samples with higher ADA titer (≥256); most of the samples with lower ADA titer (≤64) tested negative for NAbs (Supplemental Table 2).

**Fig. 3:**
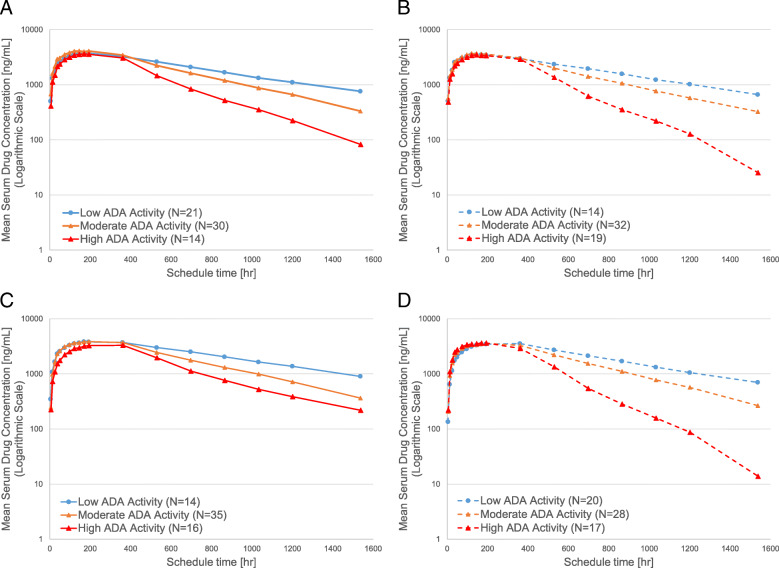
Mean serum drug concentration-time profiles (logarithmic scale) by anti-drug antibody titer in Study 1 and Study 2. (**A**) Study 1, FKB327; (**B**) Study 1, RP; (**C**) Study 2, FKB327; (**D**) Study 2, RP. ADA, anti-drug antibody. Titer subgroups: Low ADA activity (titer value of ≤25th percentile), Moderate ADA activity (titer value between 25th and 75th percentile), and High ADA activity (titer value of ≥75th percentile) at the last sampling time point

The effect of ADA activity on adalimumab pharmacokinetics was analyzed by dividing samples into three ADA titer subgroups: those with low ADA (titer value of ≤25th percentile), moderate ADA (titer value between 25th and 75th percentile), and high ADA (titer value of ≥75th percentile) (Fig. 3). Mean serum concentrations of the study drugs divided by ADA titer demonstrated a rapid decrease that was similar in both FKB327 and RP subgroups with higher ADA activity after Day 16 (360 h), when ADA activity was detected.

## Discussion

Several biosimilar agents for the adalimumab RP have been developed, and PK similarity with the RP have been confirmed for these agents [12, 19–22]. The PK similarity, as well as physicochemical and biological activity, of FKB327 has been demonstrated in comparison with the US-approved and EU-licensed RP originator in accordance with US and EU guidelines [9, 10, 12]. Some countries have issued their own biosimilar guidelines, with recommendations regarding biosimilarity assessment by totality of evidence based on scientific principles that are generally in line with those of the European Medicines Agency and US Food and Drug Administration [23]. However, additional recommendations were published in Japan, including those for analytical and functional similarity comparison using an RP marketed in the country and/or a requirement for a clinical study including patients from within the country [15, 24].

Study 1 and Study 2 were designed to assess the PK similarity, safety, and immunogenicity of the proposed biosimilar FKB327 and US-approved RP in healthy Japanese male participants as the most sensitive population to detect differences of PK and immunogenicity under no immunosuppressive medications or underlying disease conditions, and to fulfill country-specific guidelines [15, 24]. The Japanese biosimilar guidelines allow the use of an RP approved in other countries of the International Council for Harmonisation of Technical Requirements for Human Use based on clinical similarity evaluation [24]. The single-blind design of these studies is a limitation for the safety evaluation of FKB327. Study 1 and Study 2 showed similar serum drug concentration-time profiles and PK parameters for both FKB327 and the RP, supporting PK similarity between both drugs, as previously reported [12]. However, in Study 1, in which participants were administered the drug in the abdomen, AUC_0-t_, which was one of the primary PK parameters, was slightly outside the predefined bioequivalence criteria of 0.80 to 1.25, although C_max_ was within the criteria. This difference may be due to the impact of larger variability of AUC_0-t_ than C_max_ in both studies and different point estimates in Study 1, which may be a result of potentially different immunogenic backgrounds of each enrolled subject [25, 26].

It has been reported that absorption is slow, taking approximately 4 to 7 days to reach C_max_ [2], and incomplete absorption and interindividual variability of serum drug concentration after 40 mg SC injection varies due to potential lymphatic drainage compared with intravenous administration such as with infliximab [4, 27]. Therefore, Study 2 was conducted with a modified study design, which changed the administration site from the abdomen in Study 1 to the thigh to minimize absorption variability of the drugs based on the results from a previous PK comparability study using FKB327 devices dosed at the abdomen or thigh in healthy participants [20].

Although the administration site was changed in Study 2, no change was made in inclusion/exclusion criteria and number of participants compared to Study 1, and all PK endpoints were demonstrated to be within biosimilarity range with reference to the general bioequivalence criteria. A previous RP biosimilar study also reported low variability in C_max_ and reflected absorption of drug and AUC_0-∞_ accordingly in participants who received SC injections in the thigh versus in the abdomen [28]. In that study, PK endpoints of the RP biosimilar administered via SC injection to the abdomen were more variable and influenced by the amount of SC fat [28]. In our studies, the CV of the C_max_ of FKB327 and the RP were lower in variability and consistent with the other reports. Although a study with golimumab, another anti-TNF-α inhibitor, demonstrated no meaningful clinical difference in the PK of drug administered in the abdomen, thigh, and upper arm, it did show slightly higher absorption (C_max_) when administered in the thigh than in the abdomen and upper arm [29]. A study with mepolizumab also supported flexibility for the SC injection sites; however, the current study has a limitation that bioavailability and variability for injection sites may differ depending on the individual monoclonal antibody drugs [30]. Therefore, although variable PK does not have a meaningful impact on the effects of the drug [13], administration site is a factor worth considering when designing a PK equivalence study for a biosimilar product, which can show variable PK by SC injection, in order to avoid large sample sizes.

Biosimilar drug studies use highly sensitive ADA assay methods; initial studies with the RP using conventional ADA assay methods showed lower incidences of ADAs [8]. It has been reported that genetic background, such as participant polymorphisms, may influence ADA formation among those treated with anti-TNF-α inhibitors [31, 32]. Our studies detected ADA formation in most subjects who received FKB327 and RP; however, ADA titer and pattern of NAbs were similar between FKB327 and RP in both studies. Serum drug concentration-time profiles by ADA categorical titers at the last sampling time point were consistent with previous reports in that an increase in ADA activity increased the elimination rate of drug from systemic circulation [12, 20]. As described above, the ADA and ADA titer profiles between FKB327 and the RP were similar in both studies. Thus, ADA formation is considered of minimal impact on biosimilarity evaluation between studies.

In Study 2, ANCOVA included trial site, body weight, and age as covariates, using the corrected value by protein content of drugs for the statistical analysis of primary PK similarity. All primary PK parameters were within the predefined PK equivalence criteria of 0.80 to 1.25 by both ANCOVA and ANOVA, regardless of correction by protein content of drugs used in the study. Therefore, the statistical method did not impact the equivalence evaluation in Study 2. The proportions of participants who experienced ≥1 TEAE were comparable between the treatments but were slightly higher in the RP group than in the FKB327 group. All TEAEs reported were mild or moderate in intensity. The most common TEAEs and the most common AEs overall were nasopharyngitis and injection-site reaction, which are known events associated with study drug treatment, and occurred similarly between treatment groups in both studies. Overall, no clinically significant difference was seen between treatment groups in terms of safety parameters, and no new safety signals were identified in these studies.

## Conclusions

The results of Study 1 and Study 2 confirmed the PK of FKB327 to be similar to that of the adalimumab RP after a single 40-mg SC injection in healthy Japanese male participants. Immunogenicity, along with its impact on PK and the safety profile, was confirmed to be similar between FKB327 and the RP in this population.

## Supplementary Information

**Additional file 1.**

## Data Availability

The datasets used and/or analyzed during the current study are available from the corresponding author on reasonable request.

## Abbreviations

ADA: Anti-drug antibody
AE: Adverse event
ANCOVA: Analysis of covariance
ANOVA: Analysis of variance
AUC_0-t_: Area under the concentration-time curve from time zero to the last measurable concentration
AUC_0-∞_: Area under the concentration-time curve from time zero to infinity
C_max_: Maximum concentration
CI: Confidence interval
CV: Coefficient of variation
ECGs: Electrocardiograms
ECL: Electrochemiluminescence
ICH-GCP: International Conference on Harmonisation Good Clinical Practice
MSD: Meso Scale Discovery
NAbs: Neutralizing antibodies
PK: Pharmacokinetics, pharmacokinetic
RP: Reference product
SC: Subcutaneous
TEAE: Treatment-emergent adverse event
TESAE: Treatment-emergent serious adverse event
TNF: Tumor necrosis factor
VAS: Visual analog scale
