# Supplementary material for: Comparison of two biosimilarity studies of FKB327 with the adalimumab reference product: randomized phase 1 studies of single-blind, single-dose subcutaneous injection in healthy Japanese male participants
Source: BMC Pharmacol Toxicol. 2022 Jan 8;23:6. doi: 10.1186/s40360-021-00545-3 (PMC8742919; doi:10.1186/s40360-021-00545-3)
Supplement: Supplementary file 1 — Additional file 1. [file 40360_2021_545_MOESM1_ESM.pdf]

Supplemental Table 1. time course of serum concentration and anti-drug antibody status after treatment with FKB327 or the reference product in Study 1 and Study 2

	Study 1						Study 2					
	Serum drug concentration, ng/mL (CV)		ADA positive (%)		ADA titer (median, Q1, Q3)		Serum drug concentration, ng/mL (CV)		ADA positive (%)		ADA titer (median, Q1, Q3)	
Day/ time (hours)	FKB N=65	RP N=65	FKB N=65	RP N=65	FKB N=65	RP N=65	FKB N=65	RP N=65	FKB N=65	RP N=65	FKB N=65	RP N=65
Day 1, predose	0.00 (–)	0.00 (–)	5/65 (7.7)	3/65 (4.6)	0.0625 (0.0625, 0.0625)	0.0625 (0.0625, 0.0625)	0.00 (–)	0.00 (–)	5/65 (7.7)	5/65 (7.7)	0.0625 (0.0625, 0.0625)	0.0625 (0.0625, 0.0625)
Day 16, 360	3290 (19.4)	3000 (23.3)	26/65 (40.0)	25/65 (38.5)	0.0625 (0.0625, 32)	0.0625 (0.0625, 40)	3600 (16.4)	3270 (17.5)	45/65 (69.2)	39/65 60.0)	8 (0.625, 240)	4 (0.625, 200)
Day 30, 696	1610 (50.5)	1300 (60.1)	59/65 (90.8)	60/65 (92.3)	160 (40, 800)	200 (48, 800)	1770 (53.2)	1460 (56.7)	65/65 (100)	63/64 (98.4)	400 (60, 1600)	360 (48, 2400)
Day 65, 1536	417 (89.3)	310 (105.5)	64/65 (98.5)	65/65 (100)	480 (160, 1600)	800 (180, 2400)	443 (96.8)	334 (108.4)	65/65 (100)	65/65 (100)	1280 (400, 2400)	800 (200, 3200)

CV, coefficient of variation; RP, reference product; ADA, anti-drug antibody.

Supplemental Table 2. Anti-drug antibody titer and neutralizing antibodies after treatment with FKB327 or the reference product in Study 1 and Study 2

ADA titer category	Neutralizing Ab							
	Study 1				Study 2			
	NAb negative, N (%)		NAb positive, N (%)		NAb negative, N (%)		NAb positive, N (%)	
	FKB327 N=64	RP N=65	FKB327 N=64	RP N=65	FKB327 N=65	RP N=65	FKB327 N=65	RP N=65
0.0625	NA	NA	NA	NA	NA	NA	NA	NA
0.25	0	0	0	0	0	0	0	0
1	0	0	0	0	0	0	0	0
4	0	0	0	0	0	0	0	0
16	1 (1.6)	2 (3.1)	0	0	2 (3.1)	1 (1.5)	0	0
64	6 (9.4)	1 (1.5)	2 (3.1)	4 (6.2)	2 (3.1)	2 (3.1)	0	0
256	2 (3.1)	3 (4.6)	12 (18.8)	11 (16.9)	1 (1.5)	4 (6.2)	6 (9.2)	11 (16.9)
1024	0	0	16 (25.0)	17 (26.2)	0	1 (1.5)	16 (24.6)	18 (27.7)
4096	0	0	19 (29.7)	24 (36.9)	0	0	29 (44.6)	15 (23.1)
16384	0	0	5 (7.8)	3 (4.6)	0	0	7 (10.8)	11 (16.9)
65536	0	0	1 (1.6)	0	0	0	2 (3.1)	2 (3.1)
Total	9/64 (14.1)	6/65 (9.2%)	55/64 (85.9)	59/65 (90.8)	5/65 (7.7)	8/65 (12.3)	60/65 (92.3)	57/65 (87.7)

ADA, anti-drug antibody; NAb, neutralizing antibody; RP, reference product.
